# The Perils of Picky Eating: Dietary Breadth Is Related to Extinction Risk in Insectivorous Bats

**DOI:** 10.1371/journal.pone.0000672

**Published:** 2007-07-25

**Authors:** Justin G. Boyles, Jonathan J. Storm

**Affiliations:** 1 Center for North American Bat Research and Conservation, Department of Ecology and Organismal Biology, Indiana State University, Terre Haute, Indiana, United States of America; 2 Department of Ecology and Organismal Biology, Indiana State University, Terre Haute, Indiana, United States of America; University of St. Andrews, United Kingdom

## Abstract

Several recent papers evaluate the relationship between ecological characteristics and extinction risk in bats. These studies report that extinction risk is negatively related to geographic range size and positively related to habitat specialization. Here, we evaluate the hypothesis that extinction risk is also related to dietary specialization in insectivorous vespertilionid bats using both traditional and phylogenetically-controlled analysis of variance. We collected dietary data and The World Conservation Union (IUCN) rankings for 44 Australian, European, and North American bat species. Our results indicate that species of conservation concern (IUCN ranking near threatened or above) are more likely to have a specialized diet than are species of least concern. Additional analyses show that dietary breadth is not correlated to geographic range size or wing morphology, characteristics previously found to correlate with extinction risk. Therefore, there is likely a direct relationship between dietary specialization and extinction risk; however, the large variation in dietary breadth within species of least concern suggests that diet alone cannot explain extinction risk. Our results may have important implications for the development of predictive models of extinction risk and for the assignment of extinction risk to insectivorous bat species. Similar analyses should be conducted on additional bat families to assess the generality of this relationship between niche breadth and extinction risk.

## Introduction

A common goal of conservation biology is to determine the ecological characteristics that relate to a species' risk of extinction. Previous studies have shown that a species' risk of extinction may be related to characteristics such as geographic range size [1], community structure [2], dispersal ability [3], predation [4], and parasitism [5]. These studies have implications for the construction of predictive extinction models [6] and the assignment of extinction risk classifications [7], as well as in making knowledgeable conservation decisions.

One characteristic that may influence extinction risk, diet, has been studied with respect to both trophic level [8]–[10] and niche breadth [10], [11]. Rarity and extinction risk are positively correlated with trophic level in some taxa [8], [9], [12], [13], but patterns of extinction risk within trophic levels seem less clear [10], [11], [14], [15]. Differences in diet between species, specifically the level of dietary specialization, may relate to extinction risk because dietary specialists should be more sensitive than generalists to the loss of prey [16] or the destruction of prey habitat [13].

Bats (Order Chiroptera) are a widespread, ecologically diverse group comprised of over 1000 species [17], which makes them a good model for studies relating ecological and morphological characteristics to extinction risk [1], [8], [11]. Approximately 25% of bat species are of conservation concern, with a further 21% being classified as near threatened [17]. Bats are susceptible to endangerment and extinction because of a low reproductive output, habitat loss, and persecution from humans [17], so determining the ecological characteristics that exacerbate their extinction risk may be of importance to bat conservation.

Safi and Kerth [11] analyzed dietary breadth as a correlate of extinction risk in insectivorous bats and reported no relationship between diet and The World Conservation Union (IUCN) ranking of a species. They suggested that diet may not correlate with extinction risk in insectivorous bats because fecal analysis, the most common method of diet assessment in bats, may not be precise enough to elucidate the level of dietary specialization. Moreover, they suggested that diet may be less important than life-history and ecological traits (e.g., level of habitat specialization) in determining a species' extinction risk.

Here, we reexamine the relationship between dietary breadth and extinction risk in bats using different analytical techniques and a larger, yet more conservative, dataset than Safi and Kerth [11]. They used phylogenetically independent contrasts [18] and a regression approach, which are useful for determining variables that are correlated with extinction risk. However, independent contrasts are most appropriate for the analysis of continuous variables [18], although it is possible to analyze discrete variables. Safi and Kerth [11] assumed that IUCN rankings were continuous, with each ranking being equally spaced along the extinction-risk continuum. This assumption has been made in other papers [1], [9], but it is difficult to justify that IUCN rankings are evenly spaced because several unrelated species characteristics are used when assigning ranks [7]. It is also difficult to justify that all species within each ranking occupy the same space on the extinction-risk continuum, so the extinction risk variable can be best classified as categorical. Therefore, we used traditional and phylogenetically-controlled analysis of variance to test for a relationship between extinction risk and dietary breadth [3]. This method is well suited to analyzing differences within continuous variables between categorical groups [19].

We tested the hypothesis that the distribution of dietary breadths is different in each IUCN ranking. We predicted that species of conservation concern (IUCN ranking near threatened or above) are likely to have a narrower dietary breadth than are species of least concern. In addition, we tested whether dietary breadth mediates the relationship between morphological and ecological factors and extinction risk.

## Results

**Figure 1:**
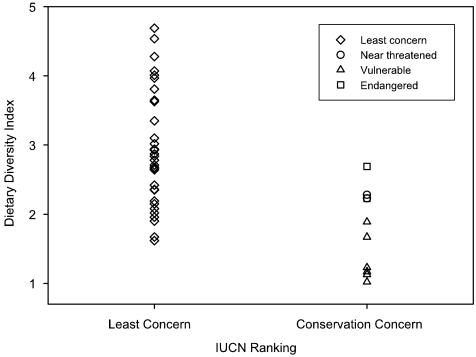
Dietary breadth as measured by a dietary diversity index (equation given in Methods) for vespertilionid bat species from Australia, Europe, and North America. Species are split into categories according to IUCN ranking.

We found no bias in the dietary diversity index (DDI) based on the number of samples collected for each species (*n* = 44; *F*_1,43_ = 0.06, *p* = 0.81). Species of least concern had an average DDI of 2.90±0.84 SD (*n* = 34) compared to 2.26±0.04 (*n* = 2) for near threatened species, 1.35±0.35 (*n* = 6) for vulnerable species, and 2.46±0.33 (*n* = 2) for endangered species (Fig. 1). Based on a traditional ANOVA, species of least concern had a significantly higher DDI than species of conservation concern (*F*_1,42_ = 15.64, *p*<0.001). When evaluated with four separate IUCN ranks, there was a significant difference in DDI between ranks (*F*_3,40_ = 6.81, *p*<0.001), although this result should be viewed with caution because of the small sample size for the near threatened and endangered rankings. However, the rankings with the large sample sizes (least concern and vulnerable) had greater variance than the rankings with the small sample sizes (near threatened and endangered), which tends to reduce the Type I error rate in an ANOVA [20]. Post-hoc Tukey tests show that species of least concern have a higher DDI than vulnerable species (*t* = 4.55, *p*<0.001), but no other pairwise comparisons are significant. No species with a DDI above 2.7 (2.7 insect orders equally represented in the diet) are of conservation concern, and 56 % (19/34) of the species of least concern have DDI values greater than 2.7. Of the species with a DDI below 2.7, 40 % (10/25) are of some conservation concern.

The difference between species of least concern and all other species remains significant after controlling for phylogeny with the Brownian (Critical F = 4.77, *p*<0.001) or Ornstein-Uhlenbeck (O-U) model (Critical F = 3.98, *p*<0.001). The difference in DDI between the four IUCN ranks also remains significant after accounting for phylogeny using either the Brownian (Critical F = 2.51, *p*<0.001) or O-U model (Critical F = 2.85, p<0.001). Based on traditional correlations and independent contrasts, DDI was not significantly correlated with body mass, aspect ratio, wing loading, or geographic range size (p>0.10 in all cases).

## Discussion

Our results suggest that vespertilionid bat species of conservation concern are more likely to be dietary specialists than are species of least concern. We found qualitatively similar results using both standard and phylogenetically-controlled analyses, as did Safi and Kerth [11], suggesting that phylogenetic inertia is relatively unimportant in explaining the relationship between diet and extinction risk. Moreover, we found no correlation between DDI and other factors related to extinction risk (e.g., wing morphology or geographic range size) in standard or phylogenetically-controlled analyses. Factors such as habitat loss, roost availability, and gregariousness undoubtedly influence the extinction risk of bat species [21], [22]. However, our analysis suggests that dietary specialization also has some direct relationship to extinction risk in vespertilionid bats. The direct application of this information by land managers may be limited because it is likely not feasible to manage the insect prey of rare bat species. However, it may be worthwhile to consider dietary specialization (along with other criteria) when evaluating the extinction risk of vespertilionid bats.

There are two possible ways of explaining the differing results between our study and Safi and Kerth [11]: the analytical techniques used and the method of data collection. Safi and Kerth [11] used independent contrasts and considered all variables to be continuous, when IUCN rankings are categorical. Furthermore, they reported dietary data collected using two methods, percent volume (%*V*) and percent frequency (%*F*) of prey types. They used regression and general linear models to convert data from both methods to the same scale (%*V*). Although we agree that percent volume and percent frequency of prey in the diet are likely related, converting between the two methods may add error to estimated DDI values. To avoid this potential problem, we limited our dataset to studies which report percent volume (%*V*) from fecal analysis. In addition, we limited our dataset to bats of the family Vespertilionidae to minimize the influence of familial differences in echolocation characteristics and wing morphology. This eliminates high duty cycle echolocating species (e.g., Rhinolopidae) and species with high aspect ratio wing morphology (e.g., Molossidae) that are represented by only a few species in Australia, Europe, and North America. We also included species from Australia, while Safi and Kerth [11] limited their dataset to Europe and North America. From our analysis alone, it is unclear if differences in analytical techniques or data collection caused the difference between our results and those of Safi and Kerth [11]. Further comparison of the studies may be useful in determining the cause of the different results as well as elucidating unforeseen patterns relating extinction risk to diet.

We have shown that vespertilionid bats of conservation concern are likely to have a narrower dietary breadth than species of least concern. The high variation of DDI within species of least concern suggests that dietary specialization may not be sufficient to cause elevated extinction risk in the absence of other factors. We argue, however, that dietary specialization may be an important characteristic to include in the evaluation of extinction risk of insectivorous bats. Unfortunately, most dietary data are from Australia, Europe, and North America and are largely limited to the Vespertilionidae. To test the generality of our results, additional analyses should be conducted when sufficient dietary data are available for other families of insectivorous bats.

## Materials and Methods

We collected dietary data for 44 species of vespertilionid bats from Australia, Europe, and North America (Table S1). We chose these regions because there are sufficient dietary and extinction risk data for bats in these areas. Data were predominantly collected from a search of the primary literature on Web of Science and Wildlife Worldwide using keywords such as diet, food habits, bat, and guano, but we also used unpublished data when published data did not exist. We included only studies that report each prey type as a percent volume (%*V*) of the entire diet using fecal analysis. Fecal analysis is a common method for quantifying the diet of insectivorous bats. This method has received some criticism because of problems caused by differential digestion and the difficulty of accurately identifying insect remains [23]. However, direct tests of fecal analysis indicate that it is useful for determining at least the relative importance of each insect order in the diet [24]. We focused on studies that report late spring and summer (approximately May to September) diet to avoid complications from seasonal variation in diet and because few studies exist on the winter diet of bats. However, some temporal variation is inherently included from studies reporting diet over an entire season. We excluded studies that assess diet using stomach content or culled prey remains to avoid variation caused by different sampling methods and studies where a large portion of the diet consisted of material other than insects (e.g., fruit, vertebrates, arthropods). Each species from a site was considered an independent sample (i.e., some studies had multiple samples). We tested for a bias based on the number of samples used to calculate the DDI of each species using a general linear model [11]. In total, we included 87 samples (range 1–8 per species) from 41 published studies and 5 unpublished data sets (Table S1).

We calculated the dietary breadth for each sample using a dietary diversity index (DDI) [25], also referred to as Levin's Index [11] or Simpson's Reciprocal Index:
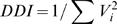
where *V_i_* represents the proportional volume of each insect order in the diet. We considered each diet to be comprised of 10 orders (Coleoptera, Lepidoptera, Hemiptera, Hymenoptera, Homoptera, Trichoptera, Diptera, Orthoptera, Neuroptera, and all other rarely represented orders, including unidentified insect remains, combined). This creates a convenient scale for DDI with 1 indicating a dietary specialist (only 1 insect order represented in the diet) and 10 indicating a dietary generalist (all 10 insect orders equally represented in the diet). We used an unweighted average of DDI's for each species in all analyses.

The conservation status of each species was obtained from The World Conservation Union's IUCN Red List of Threatened Species [26]. Species included were classified into one of four categories of risk: least concern, near threatened, vulnerable, or endangered. We first conducted an analysis comparing species of least concern with all species of conservation concern (classified as near threatened or above) grouped into one category. We then repeated the analysis considering each IUCN ranking separately [3].

We constructed a phylogenetic supertree (Figure S1) of the Vespertilionidae primarily using the mtDNA phylogeny of Hoofer and Van den Bussche [27]. Additional species were from the mtDNA and/or nuclear DNA phylogenies of Ruedi and Mayer [28], Stadelmann et al. [29], and Stadelmann et al. [30]. There was disagreement on the relationship of three species of *Vespadelus*; therefore, we performed analyses on three trees differing in the relationship of these species. Results did not differ qualitatively among trees, so we report the results based on only one tree.

We determined the relationship between IUCN rank and DDI using both traditional and phylogenetically-controlled ANOVA's. Comparative analyses were performed in the Phenotypic Diversity Analysis Program (PDAP) [31] to control for the non-independence of species. We used PDSIMUL [31] to create null *F* distributions using two models of evolutionary change: simple Brownian motion and the Ornstein-Uhlenbeck process. For each null distribution, we created 1000 simulated trees based on an initial DDI trait value of 1.0 with upper and lower bounds of 10.0 and 1.0, respectively. We used PDANOVA [31] to calculate an *F* value for each simulated tree. The difference in DDI between IUCN groups is significant at α = 0.05 if the *F* value for the traditional ANOVA is above the 95^th^ percentile of *F* values created through simulation. A complete description of this method can be found in Garland et al. [31].

To determine whether DDI is mediating the relationship between IUCN ranks and morphological or ecological variables, we performed traditional and phylogenetically-controlled correlations between these morphological and ecological variables and DDI. In general, bats with higher perceived extinction risk have small geographic ranges [1] and wing morphology suited to foraging in cluttered habitat [1], [11]. Wing morphology may be especially important in determining the dietary breadth of a species because wing morphology correlates with foraging habits [32]. A correlation between DDI and any of these variables implies that DDI may not be independently related to extinction risk. We obtained morphological (body mass, wing aspect ratio, and wing loading) and geographic range data for 33 of the 44 species included in our original analysis (Table S1). Most morphological data were taken from Norberg and Rayner [32] and geographic data were taken from Jones et al. [1]. Additional data for species unavailable in these two sources were taken from the most recent sources available. When published data did not exist, geographic range size was calculated in ArcGIS 9.2 (ESRI, Redlands, California) from digitized distribution maps. Raw correlations were analyzed on log_10_-transformed data in Statistica 6.1 (StatSoft 2003, Tulsa, Oklahoma). We transformed our comparative dataset into a set of independent contrasts using COMPARE 4.6b [33]. We used plots of absolute values of standardized independent contrasts versus their standard deviations to assess the adequacy of branch lengths.

## Supporting Information

Table S1 Dietary Diversity Index Data. Dietary breadth (DDI value) and IUCN conservation rank of insectivorous vespertilionid bats from Australia, Europe, and North America. Asterisk denotes species in COMPARE analysis.
(0.08 MB DOC)

Figure S1 Phylogenetic supertree of 44 Vespertilionid bat species from Australia, Europe, and North America used in PDAP comparative analysis. Branch lengths set equal to one.
(0.03 MB DOC)
