# Supplementary material for: The Perils of Picky Eating: Dietary Breadth Is Related to Extinction Risk in Insectivorous Bats
Source: PLoS One. 2007 Jul 25;2(7):e672. doi: 10.1371/journal.pone.0000672 (PMC1914379; doi:10.1371/journal.pone.0000672)
Supplement: Table S1 — Dietary Diversity Index Data. Dietary breadth (DDI value) and IUCN conservation rank of insectivorous vespertilionid bats from Australia, Europe, and North America. Asterisk denotes species in COMPARE analysis. (0.08 MB DOC) [file pone.0000672.s001.doc]

Table S1.

| Species | IUCN Rank | DDI | Number of Samples | References |
| --- | --- | --- | --- | --- |
| *Antrozous pallidus* | 1 | 4.01 | 1 | Easterla and Whitaker 1972. J Mammal 53:887-890 |
| *Barbastella barbastellus*^*^ | 3 | 1.23 | 3 | Rydell et al. 1996. Ecography 19:62-66; Sierro and Arlettaz. 1997. Acta Oecol 18: 91-106. |
| *Chalinolobus gouldii*^*^ | 1 | 1.90 | 1 | O’Neill and Taylor 1987. Aust J Ecol 14:19-31 |
| *C. morio*^*^ | 1 | 2.64 | 1 | O’Neill and Taylor 1987. Aust J Ecol 14:19-31 |
| *Corynorhinus rafinesquii*^*^ | 3 | 1.13 | 1 | Hurst and Lacki 1997. J Mammal 78:525-528 |
| *C. townsendii*^*^ | 3 | 1.17 | 3 | Dalton et al. 1986. Va J Sci 37:248-253; Sample and Whitmore 1993. J Mammal 74:428-435; Leslie and Clark 2002. Acta Chiropterol 4:173-182 |
| *Eptesicus fuscus*^*^ | 1 | 2.36 | 8 | Whitaker et al. 1981. Northwest Sci 55:281-292; Brigham and Saunders 1990. Northwest Sci 64:7-10; Whitaker 1995. Am Midl Nat 134: 346-360; Whitaker and Weeks 2001. Proc Indiana Acad Sci 110:123-125; Agosta and Morton 2003. Northeast Nat 10:89-104; Carter et al. 2003. Northeast Nat 10:83-88; Whitaker 2004. J Mammal 85:460-469; Whitaker and Barnard 2005. Southeast Nat 4:111-118 |
| *E. nilssoni* | 1 | 2.93 | 4 | Rydell 1986. Holarctic Ecol 9:272-276; Rydell 1989 J Zool 227:517-529; Rydell 1989. Holarctic Ecol 12:16-20; Gajdosik and Gaisler 2004. Folia Zool 53:7-16 |
| *E. serotinus*^*^ | 1 | 3.10 | 1 | Gajdosik and Gaisler 2004. Folia Zool 53:7-16 |
| *L. noctivagans*^*^ | 1 | 2.68 | 3 | Whitaker et al. 1981. Northwest Sci 55:75-77; Whitaker et al. 1981. Northwest Sci 55:281-292; Carter et al. 2003. Northeast Nat 10:83-88 |
| *L. borealis*^*^ | 1 | 2.78 | 2 | Carter et al. 2003. Northeast Nat 10:83-88; Whitaker 2004. J Mammal 85:460-469 |
| *L. cinereus*^*^ | 1 | 1.67 | 4 | Whitaker 1972. Can J Zool 50:877-883; Whitaker et al. 1981. Northwest Sci 55:281-292; Rolseth et al. 1994. J Mammal 75:394-398; Carter et al. 2003. Northeast Nat 10:83-88 |
| *L. seminolus* | 1 | 2.02 | 1 | J.O. Whitaker, unpublished data |
| *Myotis austroriparius* | 1 | 2.87 | 1 | Zinn and Humphrey 1981. Florida Scientist 44:81-90 |
| *M. blythii* | 1 | 2.35 | 3 | Arlettaz et al. 1997. J Anim Ecol 66:897-911 |
| *M. californicus*^*^ | 1 | 3.02 | 1 | Whitaker et al. 1981. Northwest Sci 55:281-292 |
| *M. ciliolabrum* | 1 | 2.15 | 1 | Whitaker et al. 1981. Northwest Sci 55:281-292 |
| *M. dasycneme*^*^ | 3 | 1.67 | 1 | Britton et al. 1997. J Zool 241:503-522 |
| *M. emarginatus* | 3 | 1.89 | 1 | Bauerova 1986. Folia Zool 35:305-310 |
| *M. evotis*^*^ | 1 | 2.71 | 1 | Whitaker et al. 1981. Northwest Sci 55:281-292 |
| *M. grisescens*^*^ | 4 | 2.70 | 2 | Rabinowitz and Tuttle 1982. Acta Theriol 27:283-293. |
| *M. leibii^*^* | 1 | 1.97 | 1 | V. Brack Unpublished Data |
| *M. lucifugus*^*^ | 1 | 4.27 | 4 | Whitaker 1972. Can J Zool 50:877-883; Whitaker et al. 1981. Northwest Sci 55:281-292; Carter et al. 2003. Northeast Nat 10:83-88; Whitaker 2004. J Mammal 85:460-469 |
| *M. myotis*^*^ | 2 | 2.23 | 7 | Arlettaz et al. 1997. J Anim Ecol 66:897-911 |
| *M. nattereri*^*^ | 1 | 2.84 | 1 | Bauerova and Cerveny 1986. Folia Zool 35:55-61 |
| *M. septentrionalis*^*^ | 1 | 3.35 | 4 | Brack and Whitaker 2001. Acta Chiropterol 3:203-210; Carter et al. 2003. Northeast Nat 10:83-88; Whitaker 2004. J Mammal 85:460-469 |
| *M. sodalis*^*^ | 4 | 2.73 | 4 | Brack and LaVal 1985. J Mammal 66:308-315; Kurta and Whitaker 1998. Am Midl Nat 140:280-286; Whitaker 2004. J Mammal 85:460-469; Tuttle et al. 2006. Northeast Nat 13:435-442 |
| *M. thysanodes*^*^ | 1 | 3.65 | 1 | Whitaker et al. 1981. Northwest Sci 55:281-292 |
| *M. velifer*^*^ | 1 | 3.97 | 1 | Marquardt unpublished data |
| *M. volans*^*^ | 1 | 1.62 | 1 | Whitaker et al. 1981. Northwest Sci 55:281-292 |
| *M. yumanensis*^*^ | 1 | 2.87 | 1 | Brigham et al. 1992. J Mammal 73:640-645 |
| *Nyctalus leisleri* | 2 | 2.28 | 2 | Waters et al. 1999. J Zool 249:173-180; Kaňuch et al. 2005. Acta Chiropterol 7:249-257 |
| *N. noctula*^*^ | 1 | 3.63 | 2 | MacKenzie and Oxford 1995 J Zool 236:322-327; Rydell and Petersons 1998. Z Saugetierkd 63:79-83 |
| *Nycticeius humeralis*^*^ | 1 | 2.94 | 3 | Whitaker and Clem 1992 Am Midl Nat 127:211-214; Whitaker 2004. J Mammal 85:460-469; Geluso et al. West N Am Naturalist *In Press* |
| *Nyctophilus geoffroyi*^*^ | 1 | 2.19 | 1 | O’Neill and Taylor 1987. Aust J Ecol 14:19-31 |
| *N. timoriensis*^*^ | 3 | 1.02 | 1 | O’Neill and Taylor 1987. Aust J Ecol 14:19-31 |
| *Perimyotis subflavus*^*^ | 1 | 4.54 | 2 | Carter et al. 2003. Northeast Nat 10:83-88; Whitaker 2004. J Mammal 85:460-469 |
| *Parastrellus hesperus*^*^ | 1 | 3.81 | 1 | Valdez and Sparks unpublished data |
| *Pipistrellus kuhlii*^*^ | 1 | 4.69 | 1 | Feldman et al. 2000. Acta Chiropterol 2:15-22 |
| *Plecotus auritus*^*^ | 1 | 4.07 | 1 | Rydell 1989. Holarctic Ecol 12:16-20 |
| *Vespadelus darlingtoni* | 1 | 2.66 | 1 | O’Neill and Taylor 1987. Aust J Ecol 14:19-31 |
| *V. regulus*^*^ | 1 | 2.42 | 1 | O’Neill and Taylor 1987. Aust J Ecol 14:19-31 |
| *V. vulturnus* | 1 | 2.08 | 1 | O’Neill and Taylor 1987. Aust J Ecol 14:19-31 |
| *Vespertilio murinus* | 1 | 1.66 | 1 | Rydell 1992. Ecography 15:195-198 |
