# Supplementary material for: The Perils of Picky Eating: Dietary Breadth Is Related to Extinction Risk in Insectivorous Bats
Source: PLoS One. 2007 Jul 25;2(7):e672. doi: 10.1371/journal.pone.0000672 (PMC1914379; doi:10.1371/journal.pone.0000672)
Supplement: Figure S1 — Phylogenetic supertree of 44 Vespertilionid bat species from Australia, Europe, and North America used in PDAP comparative analysis. Branch lengths set equal to one. (0.03 MB DOC) [file pone.0000672.s002.doc]

Figure S2.
